# Editorial: mNGS for Fungal Pulmonary Infection Diagnostics

**DOI:** 10.3389/fcimb.2022.864163

**Published:** 2022-03-11

**Authors:** Henan Li

**Affiliations:** Department of Clinical Laboratory, Peking University People’s Hospital, Beijing, China

**Keywords:** metagenomic next-generation sequencing (mNGS), fungal infection, thresholds, *Pneumocystis jirovecii*, *Candida*

In recent years, metagenomic next-generation sequencing (mNGS) has been increasingly used in clinical diagnosis and has compelling application value in the diagnosis of fungal infections. Due to the widespread use of broad-spectrum antibiotics, hormones, antitumor drugs, immunosuppressants and the extensive development of various invasive procedures, the incidence of fungal infections has increased, and fungal outbreaks have occurred in different regions. Of note, critically ill patients and patients with hematological tumors are high-risk groups for fungal infections. Fungal infections, such as pulmonary aspergillosis, invasive candidiasis, cryptococcosis, or histoplasmosis, are associated with high mortality. Most human fungal pulmonary infections are caused by opportunistic fungi, but a small number of pathogenic fungi are capable of causing infections in healthy hosts. Most opportunistic fungi are harmless, and their virulence strongly depends on the health status of the host. These opportunistic fungi are capable of causing invasive infections in immunocompromised hosts, and more interestingly, clinical cases without clear host susceptibility factors have been observed.

Rapid microbiological diagnosis of pulmonary fungal infections facilitates the timely administration of antifungal therapy. Microscopy and culture are the conventional microbiological methods used to identify fungi, but both methods are relatively insensitive, and culture is time-consuming. Histopathological diagnosis is the gold standard for invasive fungal infections. However, it is also time-consuming. Immunological detection of cell wall components in serum, such as (1,3)-β-D-glucan antigen and galactomannan antigen, has important auxiliary diagnostic value. However, there are many interference factors.

The researchers who contributed to this Research Topic presented several articles that highlight the latest advances in the understanding of the application of mNGS in the diagnosis of pulmonary fungal infections. Zheng et al. summarized recent findings on the diagnostic value of mNGS in lower respiratory tract fungal infections and the impact of different sampling methods on the detection efficiency of mNGS. mNGS can detect fungal pathogens that are difficult to diagnose using traditional detection methods. However, mNGS has no obvious diagnostic advantage in cryptococcal detection. Traditional detection methods have higher detection rates for *Candida albicans* and *Candida tropicalis* than mNGS. mNGS using bronchoalveolar lavage fluid samples is more optimal for the detection of fungi than using blood samples, but there was no significant difference in the specificity between the two sample types.

Qian et al. described pulmonary infection diagnosis with mNGS and reported inferior detection performance of mNGS for various *Candida* species. Samples were subjected to both mNGS and culturing. Culture identified 70 pathogenic bacteria or fungi in 54 samples, while mNGS identified a total of 225 pathogenic bacteria or fungi in 95 samples. These results demonstrate that mNGS has higher sensitivity than traditional culture for the detection of pathogenic bacteria and fungi (p<0.001). Nevertheless, a total of 34 bacteria/fungi which tested positive by culture were not reported by mNGS. Among these false-negative cases, 76.5% (26/34) were detected by mNGS without meeting the criteria for positive detection, while the remaining 23.5% (8/34) were not detected. In particular, mNGS showed inferior detection performance for *Candida* species. Only 21.7% (5/23) of the *Candida* species were reported by mNGS in culture-positive samples. This is possibly due to the hindrance by the commensal microbiome in the respiratory tract. Although mNGS had a better positive rate than culture in bacterial and fungal detection, its performance was highly variable across different pathogens. *Candida* species in respiratory specimens can be colonizers and may not cause pulmonary infections. Therefore, interpretation of *Candida* species in respiratory specimens still requires further research.

Chen et al. discussed the usefulness of mNGS for the diagnosis of *Pneumocystis jirovecii* pneumonia, which is generally unculturable. It causes asymptomatic or mild infections in normal hosts and fulminating pneumonia (*Pneumocystis* pneumonia) in immunocompromised hosts. In this study, fungal infections detected in seven cases by mNGS were undetectable by culture, and four of them were identified as *P. jirovecii*. Notably, among 10 kidney transplant patients, mNGS detected *P. jirovecii* in nine cases. The pathogenicity of opportunistic fungi in immunosuppressed patients is difficult to judge, and the determination of thresholds is necessary, but further research is needed.

The thresholds for pathogenic and opportunistic fungi should be different, and they should be carefully interpreted, as opportunistic fungi may simply represent colonizers or even contaminants. Incorrect identification of etiological agents would pose a major risk for unnecessary over-treatment of patients with potentially toxic drugs or harmful procedures, and/or underdiagnosis leading to poor disease management. Training should be provided to physicians to update them regarding this emerging NGS technology for laboratory diagnosis of infections, and clinical microbiologists should also be consulted when interpreting mNGS results.

Currently, fungal databases are smaller than bacterial databases, but they will gradually extend as the cost of sequencing decreases and more sequencing data are submitted. The accuracy of interpreting mNGS results based on these fungal databases should be further improved. The combination of mNGS and conventional methods will provide powerful support in the diagnosis of pulmonary fungal infections.

In conclusion, this themed collection enhances our knowledge of the application of mNGS in pulmonary infections, and the advantages and disadvantages of fungal pathogen detection by mNGS have been discussed. In the future, through more real-world research and the expansion of fungal databases, mNGS may play a greater role in fungal infection diagnosis, outbreak monitoring, and drug resistance analysis.

## Author Contributions

The author confirms being the sole contributor of this work and has approved it for publication.

## Conflict of Interest

The author declares that the research was conducted in the absence of any commercial or financial relationships that could be construed as a potential conflict of interest.

## Publisher’s Note

All claims expressed in this article are solely those of the authors and do not necessarily represent those of their affiliated organizations, or those of the publisher, the editors and the reviewers. Any product that may be evaluated in this article, or claim that may be made by its manufacturer, is not guaranteed or endorsed by the publisher.

